# Kombinationstherapien in der Immunonkologie: Differenzierte regulatorische Herangehensweisen

**DOI:** 10.1007/s00103-020-03228-2

**Published:** 2020-10-21

**Authors:** Jan Müller-Berghaus, Sinan B. Sarac, Martina Schüssler-Lenz

**Affiliations:** 1 Paul-Ehrlich-Institut, Paul-Ehrlich-Str. 51–59, 63225 Langen, Deutschland; 2 Danish Medicines Agency, Lægemiddelstyrelsen, Axel Heides Gade 1, 2300 Kopenhagen S, Dänemark

**Keywords:** Checkpoint-Inhibitoren, Arzneimittelzulassung, Klinische Entwicklung, Klinische Prüfung, Immuntherapie, Checkpoint inhibitors, Marketing authorisation, Clinical development, Clinical trial, Immunotherapy

## Abstract

Kombinationstherapien von unterschiedlichen Arzneimitteln sind fester Bestandteil der Medizin. Wissenschaftlich und regulatorisch gilt jedoch der Anspruch, den Beitrag jedes Arzneimittels zum Gesamteffekt zu verstehen. Deshalb müssen nichtklinische und klinische Entwicklungsprogramme diese Aspekte berücksichtigen und entsprechend gestaltet werden. Derzeit befinden sich viele Arzneimittel in Entwicklung, die versuchen durch Verwendung und Aktivierung von Komponenten des Immunsystems des Patienten bösartige Erkrankungen langfristig zu kontrollieren. Oft wird hier der Begriff Immunonkologie verwendet. Arzneimittel, die für die Immunonkologie entwickelt und verwendet werden, können völlig unterschiedlichen Arzneimittelklassen zugeordnet werden.

In diesem Beitrag erfolgt eine Analyse von Kombinationstherapien in der Immunonkologie mit biotechnologisch hergestellten Arzneimitteln auf Basis von regulatorischen Gesichtspunkten und Erfordernissen. Dies beinhaltet Checkpointinhibitoren, genetisch modifizierte Zelltherapien, Tumorvakzinen und onkolytische Viren. Anhand dieser heterogenen Gruppe von immunonkologischen Arzneimitteln, die in Kombinationstherapien zugelassen wurden, werden die Herausforderungen in der klinischen Entwicklung erarbeitet. Wegen der unterschiedlichen Charakteristika und Anzahl der Kombinationspartner muss für jede Entwicklung ein individuell zugeschnittenes Programm entwickelt werden, d. h., es gibt keine für alle Entwicklungen anwendbare Standardlösung.

## Abstract (en)

Combination therapies of different drugs are an integral part of medicine. However, there is a scientific and regulatory need to understand the contribution of each drug to the overall effect, i.e., nonclinical and clinical development programs have to consider these aspects and need to be designed accordingly. Many drugs are currently under development that attempt to control malignant diseases in the long term by using and activating components of the patient’s own immune system. The term immuno-oncology is often used in this context. Medicines that are developed and used for immuno-oncology can be assigned to completely different classes of medicines.

This article provides an analysis of combination therapies in immuno-oncology with medicinal products produced by biotechnological manufacturing. This encompasses checkpoint inhibitors, genetically modified cell therapies, tumor vaccines, and oncolytic viruses. The challenges in clinical development are demonstrated on the basis of this heterogenous group of approved immuno-oncological drugs that have been investigated in combination therapies. Due to the different characteristics and number of combination partners, an individually tailored program must be designed for each development program and there is no standard solution.

## Einleitung

Die Kombination von unterschiedlichen Arzneimitteln stellt eine besondere Situation für klinische Prüfung und Zulassung dar, da prinzipiell die Notwendigkeit jeder Komponente begründet werden sollte und der Beitrag der Einzelkomponenten zu Wirksamkeit und Sicherheit untersucht werden muss. Immunonkologische Arzneimittel spielen derzeit eine besondere Rolle in der Zulassung, da ein erheblicher Anteil der klinischen Entwicklung für onkologische Arzneimittel in dieser besonderen Klasse von Arzneimitteln stattfindet. Während die ersten Zulassungen in der Monotherapie erfolgten, kamen in der Folge zunehmend mehr Kombinationstherapien in die klinische Entwicklung und zur Zulassung. Auf Basis der bereits erfolgten Zulassungen von Kombinationen mit immunonkologischen Arzneimitteln wird die Herangehensweise von Entwicklern und Regulatoren dargestellt und besondere Herausforderungen werden herausgearbeitet. Da der Begriff Immunonkologie zwar recht häufig in der Literatur Verwendung findet, aber nicht exakt definiert ist, wurde für diesen Artikel eine Definition vorgenommen, wobei die zurzeit am häufigsten diskutierten immunonkologischen Arzneimittel in dieser Definition enthalten sind.

## Wissenschaftliche und regulatorische Aspekte von Kombinationstherapien

Die Kombination von Arzneimitteln findet letztlich immer mit dem Ziel statt, die klinischen Ergebnisse für die Patienten zu verbessern, sei es durch eine verbesserte Wirksamkeit oder durch weniger oder geringere unerwünschte Wirkungen. Regulatorisch ist zu unterscheiden zwischen der bloßen Kombination der Arzneimittel in der klinischen Prüfung, dann aber der Zulassung des einzelnen Produktes, und der festen Kombination von zwei oder mehreren Arzneimitteln in einem Produkt, das dann zugelassen wird. Grundsätzlich besteht die Anforderung, den Beitrag jedes Kombinationspartners zur Gesamtwirksamkeit und -sicherheit zu definieren und zu verstehen [1]. Diese Anforderung steht oft im Kontrast zu den durchgeführten Entwicklungsprogrammen für die Kombination von Arzneimitteln und ist dann Ausgangspunkt für kontroverse Diskussionen und erschwerte Entscheidungsfindung.

Die Kombination von unterschiedlichen Arzneimitteln ist seit Langem fester Bestandteil der Arzneimitteltherapie in der Onkologie. Dies beinhaltet die Kombination von mehreren zytotoxischen Arzneimitteln, die Kombination von zytotoxischen und nichtzytotoxischen Arzneimitteln und die Kombination von mehreren nichtzytotoxischen Arzneimitteln, z. B. in der Immunonkologie. Der Übergang von der einfachen Kombination von Arzneimitteln zu komplexen Therapieregimen ist fließend und stellt daher eine Herausforderung für die Arzneimittelzulassung dar, die (traditionell) eher das einzelne Arzneimittel als das Gesamtregime im Fokus hat. Ein weiterer Aspekt in der Entwicklung und Zulassung von Arzneimitteln ist, ob einzelne Partner der Kombinationstherapie bereits zugelassen sind oder ob es sich um die Co-Entwicklung von zwei oder mehr neuen Arzneimitteln handelt.

Bei der Kombination von onkologischen Arzneimitteln, sowohl konventionellen zytotoxischen Arzneimitteln, Immuntherapien und zielgerichteten Tumortherapien, sind unterschiedliche pharmakodynamische Interaktionen denkbar [2].

- Es gibt eine wirksame „Standardtherapie“ (einzelnes Arzneimittel oder Arzneimittelkombination) und es wird ein neues Medikament A entwickelt, das bei der Kombination eine deutliche Verstärkung in den nichtklinischen Modellen zeigt. Dies setzt voraus, dass die Wirkungsmechanismen und Nebenwirkungsprofile der Standardtherapie und des neuen Wirkstoffs nicht oder nur gering überlappen. Akzeptierte Praxis in der Arzneimittelentwicklung ist es, ein neues Arzneimittel zur „Standardtherapie“ hinzuzufügen. Aus regulatorischer Sicht ist dann die Erwartung, dass die Überlegenheit der Kombination (neues Arzneimittel A plus Standardtherapie C) gegenüber der Standardtherapie C durch eine zweiarmige, randomisierte klinische Prüfung belegt wird [2]. Die Notwendigkeit der Standardtherapie und der Beitrag zur Gesamtwirksamkeit werden mit diesem Konzept nicht überprüft und bleiben letztlich unbekannt. Es bleibt auch unbekannt, inwieweit eine sequenzielle Anwendung hinsichtlich Lebenszeitverlängerung und Lebensqualität besser sein könnte als die gleichzeitige Anwendung. Unterstützende Daten für die jeweiligen Hypothesen sollten jedoch supportive klinische Studien und nichtklinische Modelle liefern. Fragen zur Dosierung der Einzelkomponenten und mögliche pharmakokinetische Interaktionen müssen in explorativen Studien untersucht werden.
- Das Arzneimittel B hat alleine keine relevante Wirksamkeit, verstärkt aber relevant die Wirksamkeit des anderen Arzneimittels A. Der Beitrag des Arzneimittels B zur Wirksamkeit muss in nichtklinischen Studien gezeigt werden, eine alleinige Prüfung des Arzneimittels B zum Beleg der klinischen Aktivität in explorativen klinischen Phase-II-Studien ist nicht erforderlich. Falls es sich um die Neuentwicklung von zwei Substanzen handelt, sollte die klinische Aktivität der Kombinationstherapie A + B in einer dreiarmigen Phase-II-Studie mit A alleine und der Standardtherapie verglichen werden.
- Wenn beide Arzneimittel alleine eine moderate Aktivität, bei der Kombination aber eine deutliche Verstärkung in den nichtklinischen Modellen zeigen, sollte die vierarmige Phase-II-Studie die Einzelsubstanzen mit der Kombination und der Standardtherapie vergleichen, also A gegen B gegen A + B gegen Standardtherapie.
- Wenn in nichtklinischen Studien gezeigt wurde, dass beide Arzneimittel alleine keine relevante Aktivität haben und nur in der Kombination wirken, kann auf die Prüfung der Einzelsubstanzen in der explorativen klinischen Phase-II-Studie verzichtet werden. In diesem Fall sollte die Kombination A + B gegen Standardtherapie geprüft werden.

## Immunonkologie

In den letzten Jahren spielt ein Therapiezweig der Onkologie, die Immunonkologie, eine zunehmend wichtigere Rolle. Der Begriff Immunonkologie ist letztlich nicht eindeutig definiert. Für diesen Beitrag soll er therapeutische Strategien bezeichnen, die durch gezielte Manipulation oder Aktivierung der körpereigenen, spezifischen, zellulären Immunmechanismen für die Behandlung von malignen Erkrankungen verwendet werden. Die Therapie mit monoklonalen Antikörpern, die gegen Zielstrukturen auf den malignen Zellen gerichtet sind, wie z. B. Trastuzumab (gerichtet gegen HER2 beim Brustkrebs) oder Rituximab (gerichtet gegen CD20 beim follikulären Lymphom), ist dagegen nicht in der hier gewählten Definition der Immunonkologie enthalten. Auch Zytokine und immunomodulatorische Arzneimittel wie Thalidomid, Lenalidomid und Pomalidomid, die multiple Effektormechanismen haben, werden daher in diesem Artikel nicht berücksichtigt. Des Weiteren sollen nur biotechnologisch hergestellte, zugelassene immunonkologische Arzneimittel, die in Kombinationen verwendet wurden, betrachtet werden, weil hier die Evidenz zu Wirksamkeit und Sicherheit gut dokumentiert ist. Die Betrachtungen beschränken sich daher auf sog. Checkpointinhibitoren, Varianten der adoptiven Zelltherapien, aktive Immuntherapien („Tumorvakzine“) und onkolytische Viren.

Die sog. Checkpointinhibitoren sind die Produktklasse, die derzeit am intensivsten in unterschiedlichen onkologischen Indikationen geprüft wird. Hierzu zählen z. B. die monoklonalen Antikörper gegen CTLA‑4 (Ipilimumab) und PD‑1 bzw. PD-L1 (bereits zugelassen: Atezolizumab, Avelumab, Cemiplimab, Durvalumab, Nivolumab, Pembrolizmab), die u. a. durch Blockade eines Rückkopplungsmechanismus die körpereigene Immunantwort aktivieren.

Schon vor vielen Jahren wurden beeindruckende Ergebnisse bei einzelnen Patienten durch die Ex-vivo-Expansion von tumorspezifischen T‑Zellen mit anschließender Rückinfusion erzielt (sog. adoptiver T‑Zelltransfer). Eine antigenspezifische Variante dieser Therapie sind die durch Gentransfer mit einem spezifischen chimären Antigenrezeptor („chimeric antigen receptor“, CAR) ausgestatteten, patienteneigenen T‑Zellen (bereits zugelassen: Axicabtagene ciloleucel, Tisagenlecleucel). Der Großteil der Entwicklungen spielt sich hier in der Hämatoonkologie ab, aber auch solide Tumoren sind Gegenstand intensiver Forschungen.

Tumorvakzine, die auf der direkten, in vivo antigenspezifischen Aktivierung des Immunsystems aufbauen, werden schon seit vielen Jahren klinisch evaluiert. Hierzu gehören Immuntherapien auf Basis von Plasmiden, mRNA-Technologien, viralen Vektoren und dendritischen Zellen. Das erste zugelassene Produkt, das auf einer Impfstrategie basierte, war Sipuleucel‑T gegen das Prostatakarzinom. Hierbei handelte es sich um autologe, antigenpräsentierende Zellen des Patienten, die nach In-vitro-Differenzierung und Antigenbeladung wieder zurück an den Patienten gegeben wurden. Einige Jahre nach erfolgter Zulassung wurde vom pharmazeutischen Unternehmer die Zulassung wieder zurückgenommen.

Auch onkolytische Viren, die z. B. nach direkter Injektion in eine Tumormetastase Tumorzellen infizieren und zerstören, haben als eine weitere Komponente ihrer Wirksamkeit die Aktivierung des patienteneigenen Immunsystems. Dies kann über Effekte der Therapie auf nichtbehandelte Metastasen geschlussfolgert werden. Das zugelassene Produkt Talimogene laherparepvec ist ein gentechnisch manipuliertes Herpesvirus, das zudem für GM-CSF, ein immunstimulatorisches Zytokin, codiert.

## Gegenwärtig untersuchte Kombinationstherapien

Auf Basis unterschiedlicher Wirkungsmechanismen sind die verschiedensten Kombinationen von Immunonkologika mit anderen Arzneimitteln vorstellbar mit dem Ziel, synergistisch wirkende Therapiekonzepte zu entwickeln. Wissenschaftliche Erkenntnisse, dass Chemotherapie, Bestrahlung und Inhibition des Enzyms PARP (Poly-ADP-Ribose-Polymerase) die Antigenpräsentation erhöhen, sprechen zum Beispiel für die Kombination von Chemo- und Radiotherapie mit Immuntherapie. Die Immigration von Immunzellen in den Tumor kann durch die Beeinflussung der Tumorgefäße beeinflusst werden. Hier wird vor allem die Inhibition von vaskulären endothelialen Wachstumsfaktoren (VEGF), z. B. durch Bevacizumab und einige Tyrosinkinaseinhibitoren, untersucht. Eine tumorbedingte Immunsuppression im Tumormikromilieu aufzuheben, stellt einen weiteren Angriffspunkt für Immunonkologika dar. Auch die Kombination von unterschiedlichen Immunonkologika, wie z. B. die Kombination aus Vakzinierung und Checkpointinhibition, ist eine Option, die in Studien untersucht wird.

## Zugelassene Kombinationstherapien in der Immunonkologie

Die Tab. 1 stellt dar, welche immunonkologischen Arzneimittel in Kombination mit einem weiteren Arzneimittel zugelassen wurden. Aus Gründen der Übersichtlichkeit sind die Indikationen sehr verkürzt dargestellt und für die Kombinationspartner wurden Überbegriffe gewählt. Wie aus Tab. 1 ersichtlich, unterscheiden sich Studiendesigns und Herangehensweise, die für die zulassungsrelevanten Studien gewählt wurden, erheblich.

- Im besten Fall werden die Kombinationspartner jeweils einzeln gegen die Kombination verglichen. Sofern die Studiengröße ausreichend ist und die statistische Planung sachgerecht erfolgte, ermöglicht dies die klare Herausarbeitung des Beitrags der Einzelkomponenten zur Gesamtwirksamkeit und -sicherheit (Beispiel Ipilimumab; A vs. B vs. A + B).
- Der sicherlich häufigste Fall ist ein Studiendesign, bei dem das neue Arzneimittel zur Standardtherapie hinzugefügt wird. Wie bereits dargestellt, ist dieses Vorgehen regulatorisch akzeptiert, auch wenn spezifische Fragen zur Notwendigkeit der Einzelkomponenten nicht beantwortet werden können. Dies ist insbesondere dann augenscheinlich, wenn es sich bei der Standardtherapie bereits um die Kombination von mehreren Arzneimitteln handelt (Beispiel: Pembrolizumab). Dieser Ansatz hat Grenzen, da es nicht sinnvoll ist, immer mehr Arzneimittel zu kombinieren, ohne den Beitrag der einzelnen Komponenten zum beobachteten Gesamteffekt zu hinterfragen (A vs. A + B).
- Der Vergleich einer Kombination mit einer anderen Kombination, die als Standardtherapie gilt, wird ebenfalls regelmäßig beobachtet (Beispiele: Avelumab, Pembrolizumab). In diesen Fällen ist eine klare, wissenschaftliche Rationale für die neue Kombination, gestützt von nichtklinischen und explorativen klinischen Daten erforderlich, um diese Herangehensweise zu begründen. Wenn die Daten als nicht ausreichend erachtet werden, kann dies zur Ablehnung eines Zulassungsantrags führen, insbesondere dann, wenn die gewählten Endpunkte und das Studiendesign nicht geeignet sind, den Nutzen der Kombination zweifelsfrei zu belegen (A + B vs. C).

Die zugelassenen gentherapeutischen Arzneimittel in der Immunonkologie haben mehrere Besonderheiten.

d) Die CAR-T-Zellprodukte Axicabtagene ciloleucel und Tisagenlecleucel werden ebenfalls in Kombination mit anderen Chemotherapeutika angewendet.
  Die vor der Rückinfusion der CAR-T-Zellen durchgeführte Chemotherapiekonditionierung ist jedoch nicht zur Behandlung der Grundkrankheit gedacht und geeignet, sondern dient dazu, den CAR-T-Zellen bessere Bedingungen für die nachfolgende Proliferation zu schaffen. Diese Situation ähnelt daher dem o. g. Fall c), allerdings ist ein randomisierter Vergleich zwischen den Kombinationspartnern von vornherein nicht sinnvoll.
  Der Vergleich der Kombination mit einer anders gearteten Therapie wäre möglich. Für diese beiden Arzneimittel wurden aber keine randomisierten Studien durchgeführt, sondern es wurde eine historische Kontrolle zur Kontextualisierung hinzugezogen (A + B vs. H). Diese Herangehensweise ist risikoreich, da der therapeutische Effekt so ausgeprägt sein muss, dass er auch ohne Kontrollarm überzeugend die Wirksamkeit zeigt.
  Da die Herstellung von CAR-T-Zellen eine gewisse Zeit benötigt, kann es sein, dass zusätzliche Arzneimittel verwendet werden, um überbrückend die Erkrankung unter Kontrolle zu halten (Bridging-Therapie). Auch Art und Umfang der Bridging-Therapie sind bei der Analyse der Daten einer klinischen Prüfung genau zu analysieren, um einen relevanten Beitrag auf die Gesamtwirksamkeit auszuschließen.
e) Talimogene laherparepvec ist bemerkenswert, da es sich im Prinzip um eine Kombinationstherapie innerhalb eines Arzneimittels handelt, obgleich es regulatorisch als ein Arzneimittel gehandhabt wird. Das onkolytische Virus codiert durch genetische Modifikation auch für das immunstimulatorische Zytokin GM-CSF. Die klinische Prüfung erfolgte im Vergleich gegen GM-CSF, das auch als Arzneimittel verfügbar ist. Formal handelt es sich somit um den Vergleich der Kombination zu einem der beiden Kombinationspartner. Die Wirksamkeit der lokal administrierten GM-CSF-Monotherapie in der untersuchten Indikation war und ist nicht bekannt. Ebenso ist es unbekannt, ob lokal administriertes oder lokal exprimiertes GM-CSF eine unterschiedliche Wirkung haben könnten (A + B vs. B′).

**Table 1:** 

Substanz	Kombinationspartner	Indikation	Studiendesign, durchgeführter Vergleich	Vergleichsmodell	Referenz
Atezolizumab (A)	Zytotoxische Chemotherapie (C)	Mammakarzinom	Zweiarmig, randomisiert, Kombination gegen Kombinationspartner alleine (Standardtherapie)	A + C vs. C	[7]
Avelumab (A)	Tyrosinkinaseinhibitor (B)	Nierenzellkarzinom	Zweiarmig, randomisiert, Kombination gegen Standardtherapie (C)	A + B vs. C	[8]
Ipilimumab (A)	Zweiter Checkpointinhibitor (Nivolumab; B)	Malignes Melanom	Dreiarmig, randomisiert, Kombination gegen jeweilige Einzelsubstanz	A vs. B vs. A + B	[4]
Ipilimumab (A)	Zweiter Checkpointinhibitor (Nivolumab; B)	Nierenzellkarzinom	Zweiarmig, randomisiert, Kombination gegen Standardtherapie (C)	A + B vs. C	[5]
Pembrolizumab (A)	Zytotoxische Chemotherapie (C)	Nichtkleinzelliges Bronchialkarzinom	Zweiarmig, randomisiert, Kombination gegen Kombinationspartner alleine	A + C vs. C	[9]
Pembrolizumab (A)	Zytotoxische Chemotherapie (C)	Plattenepithelkarzinom der Kopf-Hals-Region	Dreiarmig, randomisiert, Kombination gegen Kombinationspartner alleine, zusätzlicher Monotherapiearm	A vs. A + C vs. C	[10]
Pembrolizumab (A)	Tyrosinkinaseinhibitor (B)	Nierenzellkarzinom	Zweiarmig, randomisiert, Kombination gegen Standardtherapie (C)	A + B vs. C	[11]
Axicabtagene ciloleucel (A)	Konditionierende Chemotherapie (B)	Diffus großzelliges B‑Zelllymphom	Einarmig	A + B vs. H	[12]
Tisagenlecleucel (A)	Konditionierende Chemotherapie (B)	Diffus großzelliges B‑Zelllymphom	Einarmig	A + B vs. H	[13]
Tisagenlecleucel (A)	Konditionierende Chemotherapie (B)	Akute lymphatische Leukämie	Einarmig	A + B vs. H	[13]
Talimogene laherparepvec (A)	Viruscodiertes GM-CSF (B)	Malignes Melanom	Zweiarmig, randomisiert, „Kombination“ gegen Kombinationspartner alleine	A + B vs. B′	[14]

Bemerkung: A, B stehen für neue Arzneimittel, C steht für eine Standardtherapie; B′ bedeutet, dass es sich nicht um das identische Arzneimittel handelt, H steht für historische Kontrolle

## Fallbeispiele Checkpointinhibitoren

Im Folgenden werden einzelne Entwicklungen detaillierter beschrieben, um die Herausforderungen bei Entwicklung und Bewertung besser darzustellen.

Im Jahr 2011 erfolgte mit Ipilimumab (Anti-CTLA4) die erste Zulassung eines Checkpointinhibitors für Patienten mit fortgeschrittenem malignen Melanom [3]. Schon in dieser ersten Zulassung traten typische Herausforderungen bei der Planung, Durchführung und Bewertung von Kombinationsstudien zutage. In diesem Fall waren dies die Wahl und der Beitrag der Arzneimittel, die für die Kombinationsstudien gewählt wurden. Die Zulassung basierte auf 2 Kombinationsstudien. Eine Studie verglich die Kombination von Ipilimumab und Vakzinierung mit gp100-Peptiden (ein Tumorantigen) mit der Kombination von Placebo und gp100 mit der Ipilimumab-Monotherapie. Die Vakzinierung mit gp100 wurde in der Studie vorgenommen, da es von den Behandlern als inakzeptabel betrachtet wurde, die Patienten ohne therapeutische Intervention in diese Studie zu rekrutieren. Es wurde die überlegene Wirksamkeit der Kombination von Ipilimumab/gp100 und der Monotherapie mit Ipilimumab gegenüber der gp100-Therapie gezeigt. Es zeigte sich kein relevanter Unterschied in der Wirksamkeit der Kombination gegenüber der Monotherapie mit Ipilimumab. Diese Studie wurde nur mit Patienten, die HLA-A2-positiv waren, durchgeführt. Dies war erforderlich, da die gewählten Epitope des Tumorantigens gp100 im Kontext von HLA-A2 präsentiert werden. Somit war formal nur die Wirksamkeit in HLA-A2-positiven Patienten belegt, andererseits gab es keine Hinweise darauf, dass der Wirkungsmechanismus von Ipilimumab auf HLA-A2-positive Patienten beschränkt sein sollte. Die Wirksamkeit der gp100-Vakzinierung war und ist unbekannt, letztlich könnte eine alleinige Vakzinierung mit gp100 sogar einen negativen Effekt haben. Dies wurde aber als unwahrscheinlich erachtet und die gp100-Vakzinierung wurde regulatorisch als „Pseudoplacebo“ eingeordnet.

Eine weitere Studie verglich die Kombination von Ipilimumab mit Dacarbazin, einem Chemotherapeutikum, mit der Kombination von Placebo und Dacarbazin. Dacarbazin wurde in dieser Studie als etablierte Standardtherapie mitgeführt, auch wenn nie zuvor gezeigt werden konnte, dass die Dacarbazin-Monotherapie zu einem verlängerten Überleben in dieser Patientenpopulation führt. Auch in dieser Studie konnte die Überlegenheit der Kombination von Ipilimumab mit Dacarbazin gezeigt werden, formal nur für die Kombination, da es keinen Monotherapiearm für Ipilimumab in dieser Studie gab.

Zusammenfassend lässt sich feststellen, dass in dem klinischen Entwicklungsprogramm für Ipilimumab Arzneimittel als Kombinationspartner ausgewählt wurden, die erhebliche Konsequenzen hinsichtlich der rekrutierten Patientenpopulation und der Beurteilbarkeit für die Anwendung in Monotherapie hatten. In einem Fall handelte es sich um eine weitere experimentelle Therapie, im anderen Fall um eine etablierte Standardtherapie, für die es jedoch keinen überzeugenden Wirksamkeitsnachweis gab. In Zusammenschau aller dieser Ergebnisse erfolgte die Zulassung in der Monotherapie, um den Patienten eine Therapie nicht vorzuenthalten, die einen Überlebensvorteil gezeigt hatte.

Ipilimumab wurde auch in Kombination mit Nivolumab, einem anderen Checkpointinhibitor bei Patienten mit fortgeschrittenem malignen Melanom geprüft. Sowohl Nivolumab als auch Ipilimumab waren bereits bei Patienten mit fortgeschrittenem malignen Melanom zugelassen. Die klinische Prüfung verglich die jeweiligen Einzelsubstanzen mit der Kombinationstherapie. Das Ergebnis war, dass die Kombinationstherapie das Überleben gegenüber den jeweiligen Einzelsubstanzen verlängerte. Es gab zudem einen numerischen Vorteil von Nivolumab gegenüber Ipilimumab bezüglich des Überlebens. Dieses Studiendesign ermöglichte eine differenzierte Beurteilung der Wirksamkeit der Einzelsubstanzen und der Kombination. Bei der Untersuchung der Subgruppen, die von der Therapie profitieren, ergab sich ein starker Hinweis, dass anscheinend nur solche Patienten von der Kombinationstherapie profitierten, die eine sehr niedrige Expression (≤1 % der Zellen) des Biomarkers PD-L1 (Ligand der Zielstruktur von Nivolumab) hatten. Es war auch zu berücksichtigen, dass die unerwünschten Wirkungen der Kombinationstherapie mehr und stärker waren als in der Monotherapie. Da es sich hier um Subgruppenanalysen handelt und es zudem Hinweise für eine höhere Ansprechrate in der Kombinationstherapie gab, erfolgte aber keine Einschränkung der Population in der Indikation. Es schien aber sinnvoll, auf die eindeutige Überlegenheit der Kombination gegenüber der Nivolumab-Monotherapie nur im Fall einer niedrigen PD-L1-Expression in dem Indikationswortlaut der Fachinformation hinzuweisen [4].

Die bereits beschriebene Kombination von Ipilimumab und Nivolumab wurde auch in einer Studie von Patienten mit fortgeschrittenem Nierenzellkarzinom geprüft. In dieser Studie wurde diese Kombination mit der bis zu diesem Zeitpunkt akzeptierten Standardtherapie mit Sunitinib verglichen. Es gab keine Therapiearme mit den jeweiligen Einzelsubstanzen. Der primäre Endpunkt der Studie war Überleben und es konnte gezeigt werden, dass die Kombination von Nivolumab und Ipilimumab der Therapie mit Sunitinib überlegen war. Trotzdem wurde der Indikationserweiterung in erster Instanz nicht zugestimmt, da insbesondere der Beitrag von Ipilimumab zur Gesamtwirksamkeit fraglich erschien und anzunehmen war, dass die unerwünschten Wirkungen durch Ipilimumab maßgeblich zunahmen [5]. In einer vom Zulassungsinhaber beantragten Zweitbegutachtung hatte die Indikationserweiterung Erfolg, es war jedoch keine Konsensempfehlung durch den Ausschuss für Humanarzneimittel der Europäischen Arzneimittel-Agentur (EMA). Zur positiven Entscheidung führten ein Evidenztransfer von der Melanomstudie (s. oben), Hinweise auf die Aktivität der Kombinationstherapie in unterstützenden Studien und ganz maßgeblich der Nachweis der Wirksamkeit über den „harten“ Endpunkt Gesamtüberleben. Die Mehrheit des Ausschusses akzeptierte letztendlich die Unsicherheit hinsichtlich des Beitrags der Einzelkomponenten wegen der nachgewiesenen Wirkung auf das Überleben [5].

## Besondere Herausforderungen der Immunonkologie

Da viele der günstigen und ungünstigen Effekte der Immunonkologika nur mit Verzögerung auftreten, ist die Dosisfindung erschwert und muss auf Surrogatparameter zurückgreifen, die in einem zeitlich überschaubaren Rahmen beobachtet werden können. Diese Unsicherheit war z. B. sehr prominent bei der Entwicklung von Ipilimumab zu beobachten, bei der noch 2 unterschiedliche Dosierungen in unterschiedlichen klinischen Prüfungen der Phase III verwendet wurden. Da es keine relevanten Studien gab, die beide Dosierungen verglichen, ergaben sich Unsicherheiten bei der Interpretation der Daten in den Zulassungsverfahren. Konsequenz dieser Unsicherheiten war die Beauflagung von weiteren Studien mit dem Ziel, die möglicherweise überlegene Dosierung herauszuarbeiten.

Bei der Kombination von Immunonkologika und zytotoxischer Immuntherapie bestanden Bedenken, dass durch eine pharmakodynamische Interaktion (Immunsuppression durch zytotoxische Wirkung der Chemotherapie auf Immunzellen) die Wirksamkeit der Immunonkologika beeinträchtigt wird. Diese Bedenken konnten zumindest zum Teil ausgeräumt werden, da für Kombinationen aus Chemotherapie und Checkpointinhibitoren klinisch relevanter Nutzen für Patienten in bestimmten Indikationen gezeigt wurde (siehe Tab. 1). Auch für Glukokortikoide, die häufig für immunologisch bedingte Nebenwirkungen gegeben werden, konnte in nichtklinischen Modellen gezeigt werden, dass der Wirkungsverlust nicht ausgeprägt erscheint.

In Abhängigkeit von der gewählten Kombination ist mit einer Zunahme der unerwünschten Wirkungen im Vergleich zu den einzelnen Komponenten zu rechnen. Es bleibt daher eine Herausforderung, den erreichten zusätzlichen Nutzen mit dem zusätzlichen Schaden ins Verhältnis zu setzen und zu einer günstigen Nutzen-Risiko-Abwägung zu kommen. Die Wahl von klinischen Endpunkten, die allgemein akzeptiert den Patientennutzen beschreiben (Überleben), und die Wahl von Studiendesigns mit geringerem Verzerrungspotenzial (randomisiert, verblindet) werden die Entscheidungsfindung bei Zulassung und Erstattung erleichtern. Auch wenn das Konzept der Verwendung des körpereigenen Immunsystems zur Bekämpfung der bösartigen Erkrankung eine gute Verträglichkeit suggeriert, wurden in unterschiedlichsten immunonkologischen Ansätzen therapiebedingte Todesfälle beobachtet. Wenn eine klinische Prüfung den besonderen Nutzen einer Therapie über eine Verminderung der unerwünschten Wirkungen belegen soll, ist aus regulatorischer Sicht dringend auf Randomisierung, Verblindung der Studie und eine hohe Compliance der Patienten im Hinblick auf deren Teilnahme an der Erhebung von Daten zur Lebensqualität zu achten.

## Komplexe Entwicklungskonzepte

Besondere wissenschaftliche Herausforderungen können Therapieansätze darstellen, wie sie z. B. bei den Vakzinierungsstrategien verfolgt werden. Bei Vakzinierungen können z. B. unterschiedliche Arten von Antigenen mit anderen Substanzen kombiniert werden, die das Immunsystem aktivieren und helfen, eine antigenspezifische Immunantwort zu generieren. Gerade bei der Verwendung von genetisch modifizierten Vektoren zur Administration des Antigens ist es häufige Praxis, unterschiedliche Vektoren für die Erstvakzinierung und die Zweitvakzinierung zu verwenden (sog. heterologes Prime-Boost-Schema). Da es sich um 2 unterschiedliche Arzneimittel handelt, ist im Detail zu begründen, warum die Anwendung in dieser Kombination notwendig ist und, wenn beabsichtigt, warum die Kombination in einem Produkt in einer Zulassung (als ein Produkt) münden sollte. Auch wenn Adjuvanzien in Vakzinen regulatorisch als Hilfsstoffe eingeordnet werden, müssen differenzierte nichtklinische und klinische Entwicklungen durchgeführt werden, um z. B. die Art des Adjuvans und die Dosis zu begründen. Wenn aber eine Kombination mit einem bereits zugelassenen Arzneimittel in der gleichen Indikation stattfindet (z. B. einem Checkpointinhibitor), ist der Beitrag der Vakzinierung zum Therapieerfolg in der klinischen Entwicklung herauszuarbeiten (s. oben) oder zu begründen, warum dies nicht möglich und sinnvoll erscheint. Die Grenze des Machbaren ist vermutlich erreicht bei aktiv personalisierten Ansätzen, bei denen jeder Patient eine individualisierte Vakzinierung auf der Basis patientenspezifischer (Neo)Epitope erhält. Der Beitrag einzelner Epitope zur Sicherheit und Wirksamkeit einer Tumorvakzine, die patientenspezifisch hergestellt wird, lässt sich nicht mehr darstellen. Letztlich wird hier das normale Paradigma der Arzneimittelentwicklung, bei dem es um eine definierte Substanz geht, aufgebrochen und ein „Gesamtkonzept“ bzw. ein Algorithmus in der klinischen Prüfung untersucht und mit der Standardtherapie verglichen werden müssen.

## Zukünftige Entwicklungen

Wie bereits ausgeführt ist eines der wesentlichen Entwicklungsprinzipien in der Onkologie das Hinzufügen von Arzneimitteln zu einer Standardtherapie. Mit diesem Design lässt sich der Beitrag der neuen Komponenten zur Gesamtwirksamkeit und -sicherheit darstellen und belegen. Die Zahl der zugelassenen Arzneimittel in hämatoonkologischen Indikationen hat jedoch stark zugenommen. Dies stellt Entwickler und Regulatoren vor zunehmende Herausforderungen im Hinblick auf die bestmögliche Strategie zur klinischen Prüfung. Auch wenn randomisierte und kontrollierte klinische Prüfungen für die Evaluierung von Wirksamkeit und Evaluierung des Nutzen-Risiko-Verhältnisses nach wie vor als geeignet betrachtet werden, stellen sich verschiedene Fragen. Wie genau sind die Vortherapien bei Auswahl der Patienten für eine klinische Prüfung zu definieren? Wie lassen sich die Folgen der unterschiedlichen Therapiepfade und der Einfluss von unterschiedlichen Therapieabfolgen und Kombinationen für den Patienten bestimmen? Wie ist der Vergleichsarm angesichts der zunehmend komplexer werdenden Therapieregime zu wählen? Die Bearbeitung dieser Fragestellungen erfordert eine grundsätzlich andere Herangehensweise, da es nicht um den Wirkungsnachweis eines Arzneimittels geht, sondern um den Vergleich von Therapiepfaden. Einige der Fragen lassen sich wahrscheinlich in randomisierten Therapieoptimierungsstudien beantworten, insbesondere wenn es sich um Erkrankungen handelt, bei denen ein guter Therapiestandard erreicht wurde, und wenn es weniger neue Arzneimittelentwicklungen gibt. In komplexeren Therapiegebieten mit vielen Optionen erfordert dies jedoch einen erheblichen zusätzlichen Aufwand, eine wissenschaftliche Kollaboration von größeren Teilen des Gesundheitssystems, die Standardisierung von diagnostischen und therapeutischen Herangehensweisen, einen langen Zeithorizont und entsprechende Finanzierung. Wie bei einem solchen Modell mit neuen und innovativen Therapien umgegangen werden könnte, ist unklar.

Als weiteres mögliches Modell könnten die gegenwärtigen Bestrebungen dienen, im großen Maßstab Patientendaten aus dem Versorgungsalltag in digitaler Form zu sammeln, unterschiedliche Datenbanken (z. B. klinische Daten, Biomarkerdaten) zu verknüpfen und auszuwerten. Neben den technischen Voraussetzungen sind hier auch Fragen der informationellen Selbstbestimmung zu beachten. Es gibt derzeit noch relativ starke Bedenken, inwieweit diese Daten belastbare Analysen stützen können, die dann Schlussfolgerungen für nachfolgende Patientengruppen ermöglichen könnten. Dies gilt wahrscheinlich noch mehr für immunonkologische Arzneimittel, deren Wirksamkeit wahrscheinlich noch stärker von bisher nicht bekannten Charakteristika der Patienten abhängt, die Art und Umfang der immunologischen Antwort determinieren. Das Verzerrungspotenzial durch Patientenselektion könnte daher besonders hoch sein. Die randomisierte Prüfung wird daher der Goldstandard für die klinische Prüfung und die Zulassung bleiben.

## Fazit

Kombinationstherapien werden wahrscheinlich eher die Regel als die Ausnahme bei der Anwendung von immunonkologischen Arzneimitteln sein. Die Bedingungen für die klinische Prüfung und Zulassung sind in Abhängigkeit der Kombinationspartner unterschiedlich und müssen im Dialog von Entwicklern und Regulatoren angepasst werden.
